# Prevalence, antimicrobial resistance, and distribution of toxin genes in methicillin-resistant *Staphylococcus aureus* from retail meat and fruit and vegetable cuts in the United Arab Emirates

**DOI:** 10.3389/fcimb.2025.1628036

**Published:** 2025-10-03

**Authors:** Ihab Habib, Mohamed-Yousif Ibrahim Mohamed, Glindya Bhagya Lakshmi, Febin Anes, Richard Goering, Mushtaq Khan, Abiola Senok

**Affiliations:** ^1^ Department of Veterinary Medicine, College of Agriculture and Veterinary Medicine, United Arab Emirates University, Al Ain, United Arab Emirates; ^2^ ASPIRE Research Institute for Food Security in the Drylands (ARIFSID), United Arab Emirates University, Al Ain, United Arab Emirates; ^3^ Department of Medical Microbiology and Immunology, College of Medicine and Health Sciences, United Arab Emirates University, Al Ain, United Arab Emirates; ^4^ Department of Medical Microbiology and Immunology, Creighton University School of Medicine, Omaha, Nebraska, NE, United States; ^5^ Zayed Centre for Health Sciences, United Arab of Emirates University, Al Ain, United Arab Emirates; ^6^ College of Medicine, Mohammed Bin Rashid University of Medicine and Health Sciences, Dubai, United Arab Emirates; ^7^ School of Dentistry, Cardiff University, Cardiff, United Kingdom

**Keywords:** methicillin-resistant *staphylococcus aureus*, retail food, antimicrobial resistance, OneHealth, United Arab Emirates

## Abstract

**Introduction:**

Methicillin-resistant *Staphylococcus aureus* (MRSA) is an emerging foodborne hazard with significant public health implications under the One Health framework. Data on MRSA in retail foods from the United Arab Emirates (UAE) remain scarce, despite the country’s heavy reliance on diverse food supply chains. This study aimed to investigate the prevalence, antimicrobial resistance (AMR) profiles, and toxin gene distribution of MRSA in retail foods of animal and plant origin in Dubai, UAE.

**Methods:**

A total of 260 food samples—including beef, sheep, camel, and chicken meat, as well as ready-to-eat fruit and vegetable cuts—were collected from major supermarkets. MRSA screening was performed using enrichment culture, followed by dual confirmation with MALDI-TOF MS for species identification and triplex PCR targeting the mecA gene. Antimicrobial susceptibility was assessed in 87 confirmed isolates against multiple antibiotic classes. Logistic regression analysis was applied to assess associations between product forms and MRSA contamination. Detection of enterotoxin and exfoliative toxin genes was performed using PCR assays.

**Results:**

MRSA was detected in 47.7% of samples, with the highest prevalence in chicken meat (75%), followed by camel (55%) and beef (45.7%). Contamination was lower in fruit (16.7%) and vegetable cuts (30%). Minced beef exhibited significantly higher contamination (78.5%) compared to other beef forms. All 87 isolates were resistant to β-lactam antibiotics. Resistance varied across food groups for gentamicin, ciprofloxacin, erythromycin, and tetracycline. Multidrug resistance (≥3 classes) was found in 79.3% of isolates, with extensive resistance (≥4 classes) more frequent in camel (75%) and beef (65.4%) isolates. Enterotoxin genes were identified in 42.5% of isolates, predominantly sea (29.1%). Exfoliative toxin gene type A was detected most often in vegetable cuts. Dual toxin gene carriage was rare (4.6%).

**Discussion/Conclusion:**

Retail foods in the UAE, particularly chicken and camel meat, represent an important reservoir of multidrug-resistant and toxigenic MRSA. The findings highlight the One Health risks of MRSA in the food chain and underscore the need for coordinated surveillance and intervention strategies across human, animal, and environmental health sectors to mitigate transmission risks and safeguard public health.

## 1 Introduction

*Staphylococcus aureus*, a Gram-positive microorganism, naturally colonizes the skin and mucosal surfaces of approximately one-third of the healthy human population and is similarly found in various domestic and wild animals, including birds (Kadariya et al., 2014). Beyond its commensal existence, *S. aureus* is a known contaminant of various food items and is capable of causing staphylococcal food poisoning (SFP) through consuming food containing pre-synthesized enterotoxins (Léguillier et al., 2024). Staphylococcal enterotoxins (SEs) are typically grouped into two major categories: classical SEs and more recently identified non-classical forms (Shalaby et al., 2024). Among these, *sea* to *see*—classified as classical SEs—are the most commonly reported. Strains of *S. aureus* harboring genes that encode these enterotoxins and can express them under conducive conditions in food are thus considered potential foodborne pathogens (Léguillier et al., 2024; Shalaby et al., 2024).

Methicillin-resistant *S. aureus* first emerged in the 1960s and has since spread to be of global concern (Kadariya et al., 2014). Historically, MRSA was associated with nosocomial infections (HA-MRSA) in patients with co-morbidities. In the 1990s, community-associated MRSA (CA-MRSA) emerged and were associated with community acquired infections in previously healthy individuals (Enright et al., 2002; Roy et al., 2024). These CA-MRSA lineages have now evolved to be the main drivers of nosocomial infections in many regions globally. In the United Arab Emirates (UAE), national antimicrobial resistance surveillance data spanning over a decade reveals an alarming upward trend of MRSA infections from 21.9% in 2010 to 33.5% in 2021 (Thomsen et al., 2023). Notably, CA-MRSA lineages dominate clinical settings across the UAE, and their considerable genetic diversity suggests a dynamic epidemiological landscape likely driven by the country’s status as a global hub with a highly diverse population (Moradigaravand et al., 2023).

The emergence of MRSA in the food chain has garnered global attention, particularly its detection in raw and processed meat products (González-Machado et al., 2024; Khalifa et al., 2025). A systematic review revealed that MRSA was detected in 84.3% of the studies (n= 165) conducted on retail foods, even though the actual sample-level prevalence often remained below 20% (González-Machado et al., 2024). Beyond meat, fresh produce—including leafy greens, fruits, and vegetables—may also become contaminated with *Staphylococcus* spp. at various points in the food supply chain (Khalifa et al., 2025). Recently, our group reported the first isolation in the UAE of a *mecA*-positive MRSA strain belonging to sequence type ST-672 and spa type t384 from imported fresh dill (Habib et al., 2024). The principal public health concerns surrounding MRSA in food include its potential for community transmission and the risk of staphylococcal foodborne illness. In extreme cases, the ingestion of MRSA-contaminated food by a colonized or susceptible individual undergoing antibiotic treatment could allow for enterotoxin production in the gut, leading to disease (Sergelidis and Angelidis, 2017).

Despite the growing relevance of MRSA in food safety (Sergelidis and Angelidis, 2017), there remains a significant data gap in the UAE concerning its prevalence and antimicrobial resistance in meat products. This study addressed that gap by assessing MRSA prevalence in raw meats (beef, sheep meat, camel meat, and chicken) and fresh produce (ready-to-eat fruit and vegetable cuts) available in UAE retail outlets. Specifically, this work investigates the carriage of classical enterotoxin genes, evaluates antibiotic resistance patterns, and identifies the extent of multidrug resistance among MRSA isolates. This study represents the first comprehensive examination of MRSA prevalence, resistance profiles, and enterotoxin gene occurrence in retail foods of animal origin in the UAE. The findings from this study contribute valuable data to the global understanding of foodborne MRSA transmission risks, particularly from underreported regions.

## 2 Materials and methods

### 2.1 Study design and sampling

This cross-sectional study was carried out in Dubai, one of the UAE’s largest and most diverse retail markets. The sample size was calculated using an expected MRSA prevalence of 20% (González-Machado et al., 2024), with a 95% confidence level and a 5% margin of error, yielding a minimum required sample size of 246. To ensure comprehensive coverage and account for diversity in sample sources, 260 food samples were collected over six months (between September 2024 to February 2025). The sampling strategy included various food types to reflect dietary diversity and possible exposure routes. These included raw meat samples from beef (n = 70), sheep (n = 50), camel (n = 20), and chicken (n = 60), as well as ready-to-eat fresh produce items (n = 60), including fruit (n = 30) and vegetable cuts (n = 30). Red meat samples were in the form of boneless cuts, pieces with bones, and mince. Chicken meat was either whole carcasses or parts (breast, thigh, and wings) (**Table 1**). Samples were randomly collected from major supermarket outlets (n = 15) distributed across different districts in Dubai. All samples were aseptically collected in sterile containers, kept in insulated coolers with ice packs at a temperature lower than 4°C, and transported to the laboratory within 3 hours of collection to maintain microbial integrity. Upon arrival, samples were processed for microbiological analysis following standard protocols.

**Table 1:** Distribution of methicillin-resistant *Staphylococcus aureus*-positive samples across different food types (n = 260) collected from retail outlets in Dubai, United Arab Emirates.

Food type	No. of samples	No. of positive samples (%)	95% Confidence interval
Beef	70	32 (45.7)	33.7; 58.1
Sheep meat	50	22 (44.0)	30.0; 58.7
Camel meat	20	11 (55.0)	31.5; 76.9
Chicken meat	60	45 (75.0)	62.1; 85.3
Fruit cuts	30	5 (16.6)	5.6; 34.7
Vegetables cuts	30	9 (30.0)	14.7; 49.4
Total	260	124 (47.7)	41.5; 54.0

### 2.2 Isolation and identification of MRSA

A 25-gram portion from each food sample was aseptically transferred into 225 mL of Mueller–Hinton broth supplemented with 6.5% sodium chloride (HiMedia, India) to enhance selective enrichment (González-Machado et al., 2024). The inoculated broths were incubated at 37°C for 18 to 24 hours. Following incubation, a 10 µL loopful of the enriched culture was streaked onto CHROMagar MRSA (Paris, France), a chromogenic medium selective for MRSA, and incubated at 37°C for 18 to 24 hours (Diederen et al., 2005). Colonies exhibiting rose to mauve pigmentation—indicative of presumptive MRSA—were further subcultured onto nutrient agar plates to obtain pure isolates. Up to five suspect colonies per plate were selected for further identification. Species confirmation as *S. aureus* was achieved using matrix-assisted laser desorption ionization-time of flight mass spectrometry (MALDI-TOF MS), utilizing the Autobio ms1000 platform (Autobio Diagnostics, China). For DNA isolation, a commercial kit (Wizard^®^ Genomic DNA Purification Kit ((Promega, United States)) was used according to the supplier’s instructions. A multiplex polymerase chain reaction (PCR) assay targeting the *16S rRNA*, *mecA*, and *nuc* genes was performed to validate the MRSA status of the isolates, following the protocol of Maes et al. (2002) (Maes et al., 2002). Confirmed MRSA strains were then preserved in cryogenic storage at −80°C for downstream analyses.

### 2.3 Antibiotic susceptibility testing

Antimicrobial susceptibility testing of MRSA isolates was enabled using the VITEK-2 automated system (bioMérieux, France), utilizing the AST-P592 card designed specifically for *Staphylococcus*/*Enterococcus* species. A total of 15 clinically relevant antibiotics were included in the testing panel. These comprised β-lactams such as benzylpenicillin and oxacillin, with cefoxitin employed as a qualitative screen for methicillin resistance (reported as positive or negative). The panel also evaluated aminoglycoside susceptibility using gentamicin, and fluoroquinolone resistance was tested through both ciprofloxacin and moxifloxacin. Macrolide-lincosamide-streptogramin B (MLSB) resistance was assessed by including tests for erythromycin and clindamycin minimum inhibitory concentrations (MICs) alongside a qualitative screen for inducible clindamycin resistance. Glycopeptides such as teicoplanin, vancomycin, and linezolid (an oxazolidinone) were also included. Additional antimicrobials tested were tigecycline, tetracycline, rifampicin, and a combination of trimethoprim/sulfamethoxazole. Automated interpretation of MIC results was achieved using the bioMérieux VITEK-2 Advanced Expert System (AES) (bioMérieux, France). Isolates were considered multidrug-resistant (MDR) when they exhibited resistance to at least three distinct antimicrobial categories in the test panel (Magiorakos et al., 2012).

### 2.4 PCR testing for toxins encoding genes

All MRSA isolates tested for antimicrobial susceptibility testing were examined for seven toxin-encoding genes. This included screening for the five classical staphylococcal enterotoxin genes (*sea*, *seb*, *sec*, *sed*, and *see*), as well as two exfoliative toxin genes (*eta* and *etb*), using gene-specific primers (Leke et al., 2017). PCR amplification was performed on a QIAamplifier 96 thermocycler (Qiagen, Germany). The cycling protocol began with an initial denaturation at 94°C for 5 minutes, followed by 35 amplification cycles consisting of denaturation at 94°C for 2 minutes, primer annealing at 57°C for 2 minutes, and extension at 72°C for 1 minute. A final extension step was conducted at 72°C for 7 minutes to complete the reaction. The resulting PCR products were separated by electrophoresis using a 1.5% agarose gel run at 110V for 45 minutes (Leke et al., 2017). DNA bands were then visualized and documented using the GelDoc-Go imaging system (Bio-Rad, USA).

### 2.5 Statistical analysis

All statistical analyses were performed using STATA version 16.1 (StataCorp; Texas, USA). Descriptive statistics were applied to summarize the prevalence of MRSA across different food categories. Frequencies and proportions were reported and compared across MRSA-positive samples, with exact binomial 95% confidence intervals (CI) calculated to assess the precision of prevalence estimates. To investigate potential predictors of MRSA occurrence, pairwise comparisons using two-sample z-tests for proportions and logistic regression models were used. The primary outcome variable was the presence or absence of MRSA in a given food sample. Explanatory variables included food type (categorical: beef, sheep meat, camel meat, chicken meat, fruits and vegetables), and product form—stratified by commodity group. For red meat samples (beef, sheep, camel), product form was categorized as minced, bone-in cuts, and boneless cuts. The form was grouped into whole birds versus parts (e.g., wings, legs, breasts) for chicken samples. Logistic regression analyses were conducted, and odds ratios (OR) with corresponding 95% CIs were reported, and model fit was assessed using likelihood-ratio tests. A two-sided *p*-value of <0.05 was considered statistically significant for all inferential tests.

## 3 Results

### 3.1 MRSA across different food types

A total of 260 food samples were tested, of which 124 (47.7%; 95% confidence interval 41.5%; 54.0%) were positive for MRSA (**Table 1**). The highest proportion of MRSA-positive samples was observed in chicken meat (75.0%), followed by camel meat (55.0%) and beef (45.7%). Lower prevalence rates were recorded in vegetables (30.0%) and fruit cuts (16.7%) (**Table 1**).

**Figure 1:**
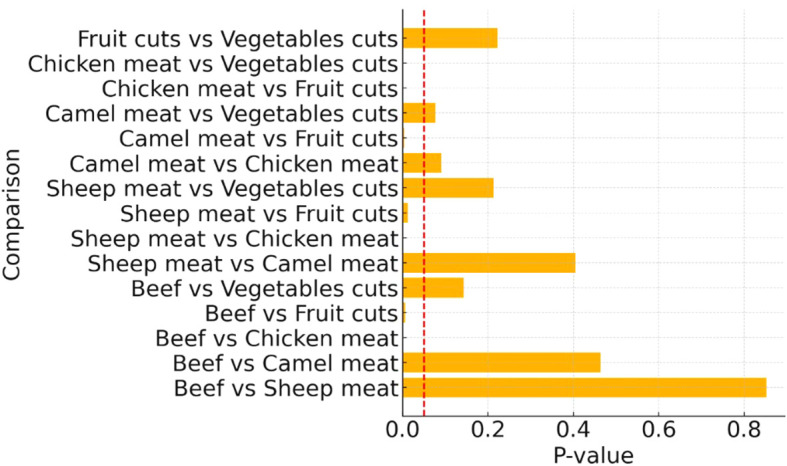
Pairwise comparison of methicillin-resistant *Staphylococcus aureus* detection in different food types. Red dashed line indicates the significance threshold at *p*-value = 0.05.

A chi-square test for independence indicated a statistically significant difference in MRSA prevalence across the different food categories (χ² = 35.46, *p* < 0.001). Pairwise comparisons using two-sample z-tests for proportions revealed that several food type comparisons had statistically significant differences in MRSA contamination levels (**Figure 1**). Chicken meat had significantly higher contamination rates than fruit cuts, vegetables, and sheep meat (*p* < 0.05 in each comparison). The differences between beef, sheep, and camel meats were less pronounced, with some comparisons approaching but not reaching statistical significance (**Figure 1**).

### 3.2 Product forms and variation in MRSA contamination

Logistic regression analysis was conducted to evaluate the association between product form and MRSA contamination across various food types (**Table 2**). Within beef products, both “pieces with bones” and “boneless cuts” were significantly less likely to be MRSA-positive compared to minced beef, which served as the reference category. Specifically, “pieces with bones” had an odds ratio (OR) of 0.04 (*p* = 0.001), and “boneless cuts” had an OR of 0.10 (*p* < 0.001), indicating a strong negative association (**Table 2**).

**Table 2:** Association between product form and methicillin-resistant *Staphylococcus aureus* contamination across different meat types based on logistic regression analysis.

Food type	Product form	Number of samples	Number of positive samples (%)	Odds ratio	*P*-value	95% Confidence interval
Beef	Mince	28	22 (78.5)	Reference category		
	Pieces with bones	14	2 (14.2)	0.04	0.001	0.007-0.261
	Boneless cuts	28	8 (28.5)	0.10	<0.001	0.032-0.369
Sheep meat	Pieces with bones	41	17 (41.4)	Reference category		
	Boneless cuts	7	2 (42.8)	1.05	0.945	0.209-5.354
Camel meat	Pieces with bones	11	8 (72.7)	Reference category		
	Boneless cuts	9	3 (33.3)	0.18	0.087	0.027-1.277
Chicken meat	Parts (breast, legs, wings)	31	23 (74.2)	Reference category		
	Whole carcass	29	22 (75.8)	1.09	0.881	0.339-3.524

In sheep meat, the odds of MRSA contamination were nearly identical between “pieces with bones” and “boneless cuts” (OR = 1.05, *p* = 0.945), suggesting no significant association between product form and contamination (**Table 2**). For camel meat, “boneless cuts” showed lower odds of MRSA contamination compared to “pieces with bones” (OR = 0.18), though this association did not reach statistical significance (*p* = 0.087) (**Table 2**). Among chicken meat samples, no significant difference in MRSA contamination was found between “parts” (breasts, legs, wings) and whole carcasses (OR = 1.09, *p* = 0.881), indicating that product form was not a predictor of MRSA presence in poultry (**Table 2**).

### 3.3 Antibiotic Susceptibility of MRSA isolates

From the 124 food samples confirmed positive for MRSA, a subset of 87 non-duplicate isolates (one isolate per positive food sample) was selected for further antimicrobial susceptibility testing and toxin gene screening. The 87 isolates were selected to be proportionally representative of MRSA isolates across different foods, distinct sampling locations, and sampling events/months.

All MRSA isolates (n = 87) from the six food categories were confirmed positive by the cefoxitin screen test (100%). High levels of resistance were also observed to benzylpenicillin and oxacillin, with near-universal resistance in the characterized isolates; only one camel meat isolate (12.5%) showed oxacillin susceptibility and a single chicken isolate (5%) was susceptible to benzylpenicillin (**Table 3**). None of the isolates exhibited non-susceptibility to linezolid, vancomycin, and teicoplanin. Only one sheep isolate (5.9%) showed resistance to rifampicin, and resistance to moxifloxacin was detected only in a single chicken isolate (5%) (**Table 3**).

**Table 3:** Antimicrobial resistance patterns of methicillin-resistant *Staphylococcus aureus* isolates (n = 87) from different food types sampled at the retail level in Dubai, United Arab Emirates.

Antimicrobial	Resistance status*	Percentage (%) of resistance per each food type					
		Beef (n = 28)	Sheep meat (n = 17)	Camel meat (n = 8)	Chicken (n = 20)	Fruit cuts (n = 5)	Vegetable cuts (n = 9)
Cefoxitin screen test	Positive	100%	100%	100%	100%	100%	100%
Benzylpenicillin	R (>=0.5)	100%	100%	100%	95%	100%	100%
	S (0.25)	—	—	—	5%	—	—
Oxacillin	R (>=4)	100%	100%	87.5%	100%	100%	100%
	S (=2)	—	—	12.5%	—	—	—
Gentamicin	R (>=16)	32.1%	11.8%	50%	15%	—	—
	I	3.6%	—	—	—	—	11.1%
	S	64.3%	88.2%	50%	85%	100%	88.9%
Ciprofloxacin	R (>=8)	78.6%	58.8%	87.5%	40%	40%	33.3%
	I	—	—	—	—	—	11.1%
	S	21.4%	41.2%	12.5%	60%	60%	55.6%
Moxifloxacin	R (>=8)	—	—	—	5%	—	—
	I	14.3%	5.9%	—	10%	—	—
	S	85.7%	94.1%	100%	85%	100%	100%
Inducible Clindamycin resistance	Negative	46.4%	58.8%	75%	80%	100%	100%
	Positive	53.6%	41.2%	20%	20%	—	—
Erythromycin	R (>=8)	67.9%	64.7%	25%	85%	—	—
	I	—	—	12.5%	—	—	—
	S	32.1%	35.3%	62.5%	15%	100%	100%
Clindamycin	R (<=0.25)	53.6%	41.2%	25%	55%	—	—
	R (>=8)	—	5.8%	—	20%	—	—
	I	—	—	—	—	—	—
	S	46.4%	53%	75%	25%	100%	100%
Linezolid	S	100%	100%	100%	100%	100%	100%
Teicoplanin	S	100%	100%	100%	100%	100%	100%
Vancomycin	S	100%	100%	100%	100%	100%	100%
Tetracycline	R (>=16)	25%	29.4%	25%	60%	60%	55.6%
	S	75%	70.6%	75%	40%	40%	44.4%
Tigecycline	S	100%	100%	100%	100%	100%	100%
Rifampicin	R (>=32)	—	5.9%	—	—	—	—
	S	—	94.1%	—	—	—	—
Trimethoprim/Sulfa	R (80)	3.6%	5.9%	12.5%	—	—	—
	R (160)	14.3%	—	37.5%	—	20%	33.3%
	R (>=320)	32.1%	35.3%	37.5%	5%	40%	33.3%
	S	50%	58.8%	12.5%	95%	40%	33.3%

*R, Resistant; S, Susceptible; I, Intermediate. The numerical values in brackets indicate resistance cut-off in µg/ml.

Resistance to gentamicin, ciprofloxacin, and erythromycin varied across the confirmed MRSA isolates from different foods. Notably, camel meat isolates exhibited the highest gentamicin resistance (50%) and ciprofloxacin resistance (87.5%), whereas sheep meat isolates showed the lowest resistance to gentamicin (11.8%) (*p* = 0.042, Fisher’s exact test). Similarly, ciprofloxacin resistance was significantly more prevalent in beef (78.6%) and camel (87.5%) compared to chicken meat (40%) and vegetable cuts (33.3%) (*p* < 0.05, chi-square test).

Inducible clindamycin resistance (ICR) was detected in 53.6% of beef isolates and 41.2% of sheep isolates but was absent in all fruit and vegetable isolates. The absence of ICR in fruit/vegetable isolates was noticeable, although not statistically significant (*p* = 0.07), compared to livestock-associated strains. Erythromycin resistance was widespread in meat isolates, particularly in chicken (85%), while fruit and vegetable isolates showed universal susceptibility (100%). Clindamycin resistance followed a similar pattern, with the highest resistance in chicken (55%) and lowest in camel (25%), while all produce isolates remained susceptible (**Table 3**).

Tetracycline resistance was moderate to high across all groups, highest in chicken (60%) and fresh produce (60% for fruit cuts; 55.6% for vegetable cuts). Trimethoprim/sulfamethoxazole resistance showed a gradient level variation, with resistance at 320 µg/mL observed in 32.1% of beef, 35.3% of sheep, and 37.5% of camel isolates. In contrast, only 5% of chicken isolates and none from fruit or vegetable samples reached this high-level resistance toward trimethoprim/sulfamethoxazole (**Table 3**).

**Figure 2:**
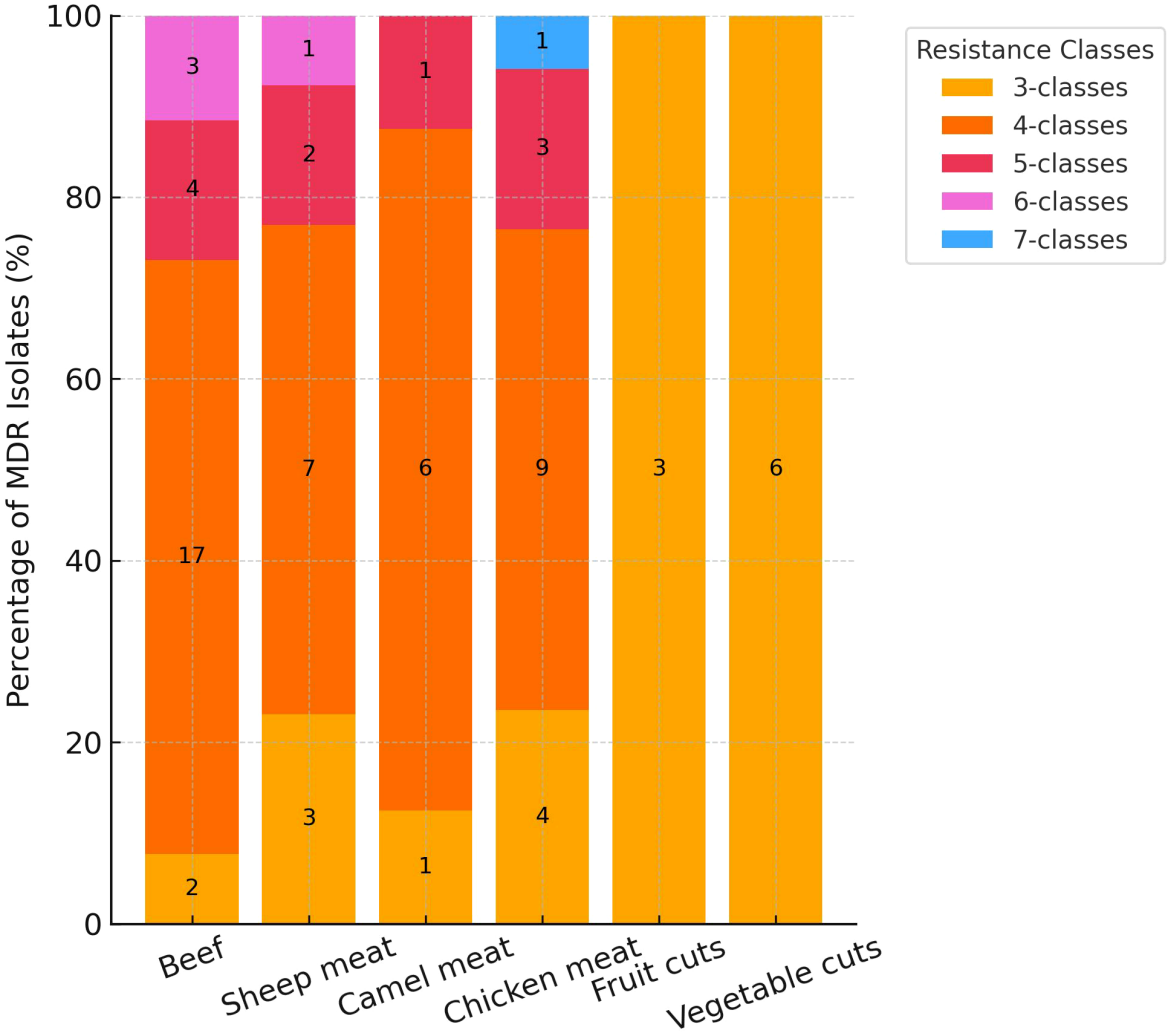
Distribution of multidrug resistance (MDR) among methicillin-resistant *Staphylococcus aureus* isolates (n = 87) by food type and number of antimicrobial classes.

The distribution of MDR among MRSA isolates varied notably across food types (**Figure 2**). Overall, 69 of 87 isolates (79.3%) were classified as MDR, exhibiting resistance to three or more antimicrobial classes. The highest proportion of extensive MDR was observed in beef and camel meat, where 65.4% and 75.0% of isolates were resistant to four or more classes (**Figure 2**). In contrast, fruit and vegetable cuts exclusively harbored isolates with resistance to only three classes. Chicken meat and sheep meat displayed intermediate patterns, with 17.7% and 15.4% of isolates, respectively, resistant to five antimicrobial classes (**Figure 2**). Notably, a small subset of chicken meat isolates (5.8%) exhibited resistance to seven classes, the highest observed in this study.

### 3.4 Toxin gene screening among MRSA isolates

**Figure 3:**
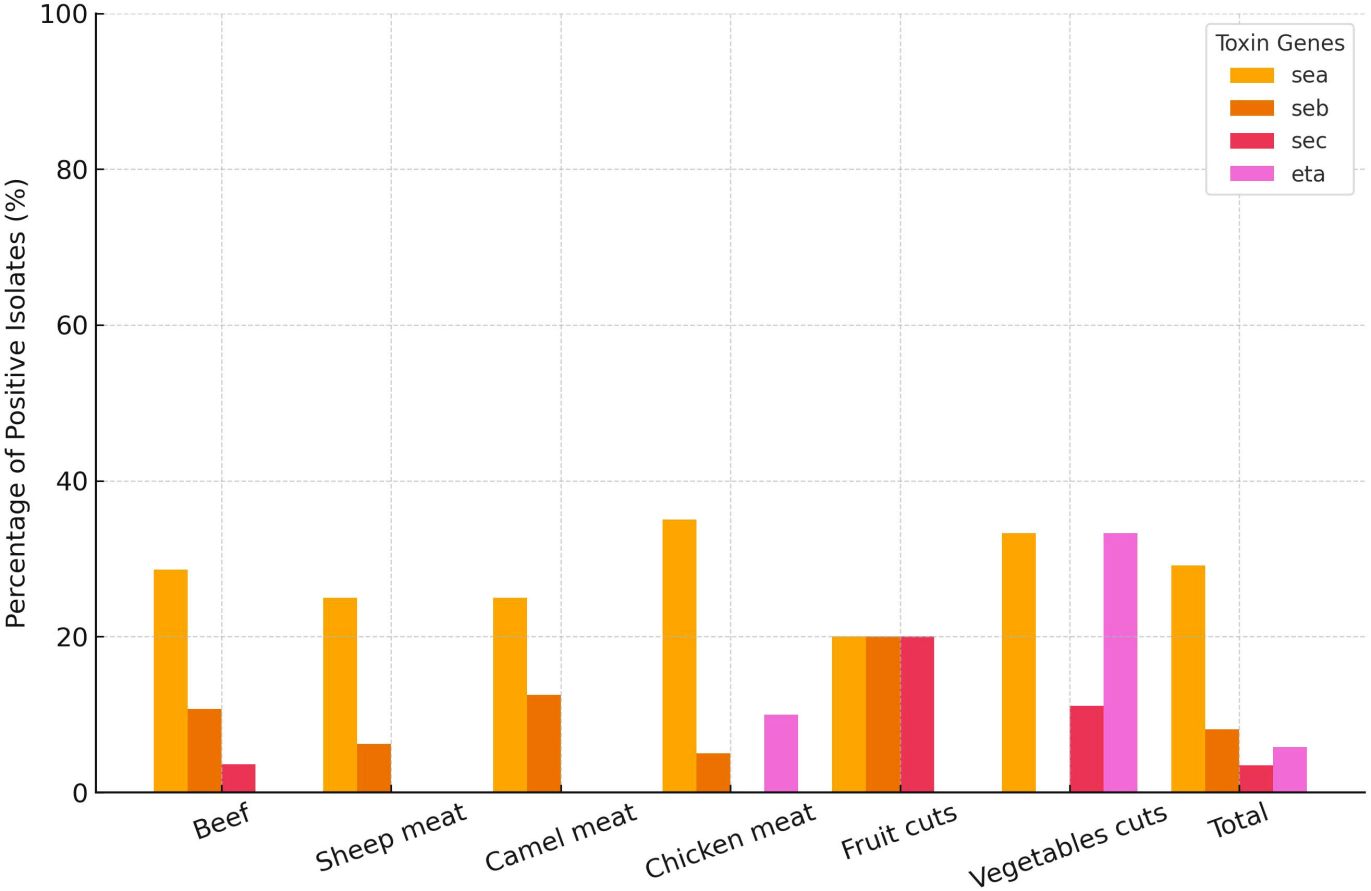
Frequency distribution of methicillin-resistant *Staphylococcus aureus* isolates (n = 87) carrying toxin genes (*sea, seb, sec*, and *eta*) across different food types sampled at retail level in Dubai, United Arab Emirates.

To assess the distribution of toxin genes among MRSA isolates (n = 87) from different food types, chi-square tests were performed for each gene. The *eta* gene showed a statistically significant variation across food categories (*χ²* = 16.61, *df* = 5, *p* = 0.005), indicating that its presence was not uniformly distributed. Further pairwise comparisons using Fisher’s exact test revealed that the prevalence of the *eta* gene was significantly higher in isolates from vegetable cuts compared to those from beef (*p* = 0.010). No other pairwise comparisons reached statistical significance. In contrast, the *sea, seb*, and *sec* genes did not differ significantly between food types (all *p* > 0.20) (**Figure 3**). Importantly, none of the isolates carried the *sed*, *see*, or *etb* genes. Overall *sea* gene was the most frequently amplified across 29.1% of all characterized MRSA isolates (**Figure 3**). A small number of MRSA isolates (n = 4 (4.6%)) were found to harbor dual toxin genes. Among beef isolates, one carried *seb* and *sec*, while another harbored *sea* and *seb*. A sheep meat isolate also carried the *seb* and *sec* combination. Notably, a single isolate from fruit cuts carried *sea* and *eta*.

## 4 Discussion

Our study provides the first comprehensive data evaluating the prevalence and characteristics of MRSA in retail foods of animal and plant origin in the UAE. This work addresses a significant data gap in the region and highlights the potential of MRSA as a foodborne pathogen. By investigating multiple food types, our findings contribute valuable baseline information to inform national food safety surveillance and One Health initiatives in the UAE.

Our findings show a relatively high overall MRSA prevalence of 47.7% in retail meat and produce samples in the UAE. Our concluded prevalence in chicken meat (75.0%) notably exceeds values reported from several regions. For instance, MRSA was detected in only 1.7% of fresh chicken meat samples in a study from China (Wang et al., 2013), whereas a prevalence of 56.3% was reported in whole chicken carcasses examined in Egypt (Elshebrawy et al., 2025). A study by Bernier-Lachance et al. (2020) in Canada found a prevalence of just 1.2%. Given that the enrichment method applied in this study was also used in comparable investigations cited herein, the higher MRSA prevalence observed in retail chicken meat samples is unlikely to be due to methodological artifacts. Instead, such variation may reflect differences in regional production systems, antimicrobial usage patterns, and biosecurity measures influencing MRSA contamination along the poultry supply chain.

Regarding beef and sheep meat, our observed MRSA prevalence of 45.7% and 44.0% is higher than some reported rates elsewhere. Studies from Canada and the United States have generally reported lower prevalence — for example, Bhargava et al. (2011) observed just 1.3% in beef, and Weese et al. (2010) found 5.6%. A report from Egypt found a rate of 4% among MRSA from beef (Hasanpour Dehkordi et al., 2017). In terms of sheep meat, our results (24% prevalence) are higher than reported in Iran (Hasanpour Dehkordi et al., 2017), and higher than the 0–5% levels reported in some European studies (González-Machado et al., 2024).

Data on camel meat MRSA prevalence is scarce globally, hence, our study strengthens the global data pool by providing novel insights into the occurrence of MRSA in camel meat, a culturally and economically important commodity in the Middle East. Compared to our finding, a Jordanian study by Quddoumi et al. (2006) found MRSA in 12.5% of camel meat samples, significantly lower than the 55% prevalence we observed. Consistent with our findings, Raji et al. (2016) and Alkuraythi et al. (2023) reported that camel meat exhibited the highest MRSA contamination rate among tested red meat types in Saudi Arabia. This suggests potential emerging risks in niche meat products in the UAE, possibly due to under-regulated handling or niche processing channels.

The logistic regression analysis strongly implicates product form as a driver of MRSA contamination in beef. Minced beef had a significantly higher prevalence (78.5%) than boneless or bone−in pieces (**Table 2**). Mincing disrupts the natural surface barrier, exposes fat and connective tissues rich in water activity, and vastly enlarges the surface area on which MRSA can adhere and multiply. It also funnels meat from multiple carcasses through common grinders and conveyors, multiplying opportunities for cross−contamination, especially if sanitation or temperature control is sub−optimal (Nastasijevic et al., 2023). Although the camel−meat regression model did not reach statistical significance (*p* = 0.087) (**Table 2**), the point estimate mirrors the beef pattern, suggesting that trimming boneless cuts could lower the bacterial load by removing surface tissue, whereas bone−in portions, which are often exposed to saw−blade aerosols, remain highly contaminated (72.7%). Given the cultural importance of camel meat in the region (Mohamed et al., 2024), this apparent reduction should not be dismissed; additional, adequately powered studies are warranted to refine the risk estimate. In contrast, chicken showed uniformly high MRSA prevalence in both whole carcasses and parts (~75%), with no difference between forms. This likely reflects the dominance of automated cutting lines in modern poultry abattoirs, where equipment surfaces, scalding tanks, and chilling baths create homogenous contamination pressure. Consequently, interventions for poultry should focus on upstream processing hygiene and equipment sanitation rather than product form only, whereas beef—and potentially camel—require distinct control points for minced versus intact cuts to mitigate consumer exposure.

Our study detected MRSA at comparatively lower rates in retail vegetable (30.0%) and fruit cut (16.7%) samples in the UAE. These findings are consistent with international reports confirming the presence of MRSA in fresh produce, albeit at varying prevalence rates. For instance, a Malaysian study found MRSA in 21.4% of leafy vegetables (González-Machado et al., 2024). A study from China also supports the potential for MRSA contamination in fresh produce, with a reported prevalence of 1.0% in vegetables and 20.5% in fresh-cut fruits and vegetables (Wang et al., 2017). In North America, MRSA was identified in 28.2% of vegetable samples in a U.S. study (Yigrem et al., 2022) and 1.4% of plant-based imports in Canada (Jung and Rubin, 2020). Although the UAE results drawn from the present study fall within this global range, detecting MRSA in raw produce remains a public health concern, especially since some of these products are consumed without cooking, bypassing a critical control point for pathogen inactivation. The findings highlight the need for enhanced hygiene practices during post-harvest handling, slicing, and retail display, as well as strengthening surveillance programs to monitor emerging trends and protect consumer health.

MRSA resistance patterns in UAE retail foods reflect concerning variation. The notably high resistance to gentamicin (50%) and ciprofloxacin (up to 88%) in camel and beef isolates suggests selective pressure from the use of aminoglycosides in camel pastoral systems and fluoroquinolones in intensive cattle farming (Nastasijevic et al., 2023; Mohamed et al., 2024). This aligns with findings from various settings, such as ciprofloxacin resistance rates of 80–100% in MRSA isolated from retail beef in Louisiana (USA) and camel meat in Riyadh (KSA) (González-Machado et al., 2024; Khalifa et al., 2025). In contrast, chicken isolates showed the highest levels of resistance to macrolides and lincosamides—particularly erythromycin (85%) and clindamycin (55%)—a pattern consistent with data from a longitudinal study in Algeria (Belhout et al., 2022), and likely driven by the historical use of these antimicrobials in poultry production. Importantly, inducible clindamycin resistance (ICR) was detected only in meat-derived isolates, particularly from beef (53.6%) and sheep (41.2%), supporting previous evidence that ICR is primarily associated with livestock sources and rarely found in plant-based foods (Endale et al., 2023). These findings highlight the importance of implementing targeted, commodity-specific antimicrobial resistance surveillance in the UAE, aligned with a One Health approach that recognizes the interconnectedness of human, animal, and environmental health.

Our findings underscore the high prevalence of MDR among foodborne MRSA isolates, with nearly 80% of isolates characterized in the present study were resistant to three or more antimicrobial classes. The presence of such broadly drug-resistant MRSA in the food chain is a significant public health concern, as it compromises treatment options for potential infections. Comparable high MDR rates in food-associated MRSA have been reported in other regions, including neighboring Saudi Arabia and beyond. For instance, a study in Riyadh found that most MRSA isolates from retail processed foods were MDR, with a notable proportion even carrying vancomycin-resistance genes (Alghizzi et al., 2021). Likewise, in Nigeria over 80% of poultry meat MRSA isolates were MDR, and a few were resistant to all tested antibiotics (Igbinosa et al., 2023). The emergence of MDR-MRSA in diverse food products has also been documented (Mohammed et al., 2025), underscoring the global nature of this threat. Collectively, these findings highlight a need for strict antibiotic stewardship in agriculture and improved food hygiene measures to curb the spread of MDR pathogens from farm to fork and protect public health.

In addition to being found in foods, enterotoxigenic MRSA strains have also been isolated from individual patients and involved in a few reported foodborne outbreaks (Sergelidis and Angelidis, 2017). Around 95% of SFP cases are linked to the classical enterotoxins *sea* to *see* (Oliveira et al., 2022). Therefore, our study focused on screening *S. aureus* isolates for these five major SE genes. In the present study, the *sea* gene was the most commonly detected in 29.1% of all characterized MRSA isolates. This aligns with findings reported in China (29.7%) (Wang et al., 2017), Malaysia (30.8%) (Puah et al., 2016) and the USA (25.8%) (Ge et al., 2017). The high prevalence of the *sea* gene in MRSA isolates from retail food in the present study is notable, given that *sea* is the most commonly linked enterotoxin in SFP cases worldwide (Sergelidis and Angelidis, 2017; Shalaby et al., 2024). On the other hand, the detection of MRSA harboring the *eta* gene—that encodes for exfoliative toxin A (ETA) which is classically associated with staphylococcal skin infections—in retail fruit− and vegetable−cut samples is epidemiologically noteworthy; *eta*−positive strains are uncommon in the wider *S. aureus* population (≈5%) but are disproportionately of human origin (Linz et al., 2023), reflecting a possible nasal or cutaneous carriage in food handlers rather than primary contamination on the farm. To minimize the dissemination of MDR and toxigenic MRSA through the food value chain, preventive measures should focus on strengthening on-farm biosecurity and enforcing prudent antimicrobial use in food animal production. In addition, strict adherence to hygiene standards during slaughter, processing, and retail handling, combined with consumer education on safe food practices, is essential to reduce the risk of transmission to humans.

MRSA isolates co-harboring MDR and toxigenic determinants have indeed been reported in various regions. For example, in Cameroon, all MRSA isolates in one study were MDR and over half carried the PVL toxin gene (Mohamadou et al., 2022). Similarly, a Pakistani study found that around 69% of *S. aureus* isolates harbored the *mecA* resistance gene while also carrying various toxin genes (e.g., the enterotoxin gene *sea* in 53.2% of isolates and the toxic shock syndrome toxin gene *tst* in 24.2%), with 42 of 62 isolates co-occurring *mecA* and multiple virulence factors (Tasneem et al., 2022). Likewise, in Egypt, the majority of MRSA isolates were reported to be MDR and simultaneously positive for toxin genes such as *sea* and PVL (Abd El-Hamid et al., 2022). The co-occurrence of MDR and toxigenic genes is alarming because it produces strains that are not only difficult to treat but also highly virulent, thereby greatly increasing the public health risk by enabling the spread of staphylococcal infections that pose significant challenges to current therapeutic strategies.

Overall, this study provides valuable insights into MRSA’s AMR profiles and toxin gene distribution in retail foods of animal and plant origin in the UAE. As one of the first efforts within a One Health framework to align MRSA surveillance across food sources in Dubai, the study contributes important baseline data to inform national risk assessments and public health interventions. Nonetheless, several limitations should be acknowledged. First, we did not collect nasal swabs or hand wash samples from food handlers at the sampling sites. While such data would have helped establish a clearer link between food contamination and food handler’s carriage, this aspect was outside the scope of the study, which focused on retail food matrices. Future studies are recommended to incorporate human sampling to better trace contamination pathways. Second, the study was geographically limited to Dubai, so it was chosen as a case study due to its diversity and relevance within the UAE and as part of an integrated ongoing One Health investigation across food, clinical, animal, and environmental sectors. While Dubai offers a rich sampling environment, extending surveillance to other emirates is important for enhancing the representativeness and generalizability of findings. Sampling from other emirates or regions where camel meat is more accessible will also ensure broader coverage of this culturally significant food item. There is also a need to conduct WGS characterization of MRSA isolates to assess their relatedness to community-acquired strains and better understand their public health significance.

## 5 Conclusion

The findings reveal a high overall prevalence of MRSA, particularly in chicken, beef, and camel meat, and to a lesser extent in fruit and vegetable cuts. The detection of key virulence genes, such as *sea* and *eta*, alongside notable resistance to antimicrobials, raises concerns about the potential role of MRSA as a foodborne pathogen and the risk of transmission through the food chain. Future research should expand geographically, include food handlers in sampling protocols, and continue exploring culturally relevant food items to better inform national food safety strategies and antimicrobial resistance mitigation plans. Although this study is regionally focused on Dubai, its findings have broader significance. The high prevalence of multidrug-resistant and toxigenic MRSA in retail foods underscores foodborne transmission risks relevant to global supply chains. Key lessons, including the need for One Health surveillance, better food hygiene, and prudent antimicrobial use, apply to other regions and support coordinated international strategies to reduce the public health threat of MRSA.

## Data Availability

The original contributions presented in the study are included in the article/supplementary material. Further inquiries can be directed to the corresponding authors.
